# Supplementary material for: Intramolecular feedback regulation of the LRRK2 Roc G domain by a LRRK2 kinase-dependent mechanism
Source: eLife. 2024 Dec 19;12:RP91083. doi: 10.7554/eLife.91083 (PMC11658767; doi:10.7554/eLife.91083)
Supplement: Figure 4—source data 1. [file elife-91083-fig4-data1.zip › Figure4SourceData1.docx]

**Detailed statistical analysis (Supplemental information for Figure 4):**

**(p values above 0.05 are shown in yellow)**

**K_M_ Anova (Std dev):**

Between: 1962784.1180 d.f. 6 Variance: 327130.6863 F: 65.5951 p:0.0000

Within: 94755.3568 d.f. 19 Variance: 4987.1240

Total: 2057539.4748 d.f. 25

**Tukey HSD Post-hoc Test:**

wt vs wt + ATP: Diff=482.5208, 95%CI=288.4480 to 676.5936, p=0.0000

wt vs R1441G: Diff=-281.8767, 95%CI=-437.4811 to -126.2723, p=0.0002

wt vs G2019S: Diff=313.7215, 95%CI=158.1171 to 469.3259, p=0.0001

wt vs K1906M: Diff=-372.2505, 95%CI=-541.6511 to -202.8499, p=0.0000

wt vs T1343A: Diff=-288.5094, 95%CI=-444.1138 to -132.9050, p=0.0001

wt vs T1343A + ATP: Diff=-225.5213, 95%CI=-381.1257 to -69.9169, p=0.0022

wt + ATP vs R1441G: Diff=-764.3975, 95%CI=-965.2819 to -563.5131, p=0.0000

wt + ATP vs G2019S: Diff=-168.7993, 95%CI=-369.6837 to 32.0851, p=0.1364

wt + ATP vs K1906M: Diff=-854.7713, 95%CI=-1066.5220 to -643.0206, p=0.0000

wt + ATP vs T1343A: Diff=-771.0302, 95%CI=-971.9146 to -570.1458, p=0.0000

wt + ATP vs T1343A + ATP: Diff=-708.0421, 95%CI=-908.9265 to -507.1577, p=0.0000

R1441G vs G2019S: Diff=595.5982, 95%CI=431.5768 to 759.6196, p=0.0000

R1441G vs K1906M: Diff=-90.3738, 95%CI=-267.5372 to 86.7896, p=0.6389

R1441G vs T1343A: Diff=-6.6327, 95%CI=-170.6541 to 157.3887, p=1.0000

R1441G vs T1343A + ATP: Diff=56.3554, 95%CI=-107.6660 to 220.3768, p=0.9113

G2019S vs K1906M: Diff=-685.9720, 95%CI=-863.1354 to -508.8086, p=0.0000

G2019S vs T1343A: Diff=-602.2309, 95%CI=-766.2523 to -438.2095, p=0.0000

G2019S vs T1343A + ATP: Diff=-539.2428, 95%CI=-703.2642 to -375.2214, p=0.0000

K1906M vs T1343A: Diff=83.7411, 95%CI=-93.4223 to 260.9045, p=0.7117

K1906M vs T1343A + ATP: Diff=146.7292, 95%CI=-30.4342 to 323.8926, p=0.1464

T1343A vs T1343A + ATP: Diff=62.9881, 95%CI=-101.0333 to 227.0095, p=0.8607

**k_cat_ Anova (Std. dev):**

Between: 0.4441 d.f. 6 Variance: 0.0740 F: 165.1621 p:0.0000

Within: 0.0085 d.f. 19 Variance: 0.0004

Total: 0.4526 d.f. 25

**Tukey HSD Post-hoc Test:**

wt vs wt + ATP: Diff=-0.2290, 95%CI=-0.2872 to -0.1708, p=0.0000

wt vs R1441G: Diff=0.0630, 95%CI=0.0164 to 0.1096, p=0.0044

wt vs G2019S: Diff=0.0020, 95%CI=-0.0446 to 0.0486, p=1.0000

wt vs K1906M: Diff=-0.0890, 95%CI=-0.1398 to -0.0382, p=0.0003

wt vs T1343A: Diff=-0.2620, 95%CI=-0.3086 to -0.2154, p=0.0006

wt vs T1343A + ATP: Diff=-0.2640, 95%CI=-0.3106 to -0.2174, p=0.0008

wt + ATP vs R1441G: Diff=0.2920, 95%CI=0.2318 to 0.3522, p=0.0000

wt + ATP vs G2019S: Diff=0.2310, 95%CI=0.1708 to 0.2912, p=0.0000

wt + ATP vs K1906M: Diff=0.1400, 95%CI=0.0765 to 0.2035, p=0.0000

wt + ATP vs T1343A: Diff=-0.0330, 95%CI=-0.0932 to 0.0272, p=0.5635

wt + ATP vs T1343A + ATP: Diff=-0.0350, 95%CI=-0.0952 to 0.0252, p=0.4983

R1441G vs G2019S: Diff=-0.0610, 95%CI=-0.1102 to -0.0118, p=0.0096

R1441G vs K1906M: Diff=-0.1520, 95%CI=-0.2051 to -0.0989, p=0.0000

R1441G vs T1343A: Diff=-0.3250, 95%CI=-0.3742 to -0.2758, p=0.0321

R1441G vs T1343A + ATP: Diff=-0.3270, 95%CI=-0.3762 to -0.2778, p=0.0328

G2019S vs K1906M: Diff=-0.0910, 95%CI=-0.1441 to -0.0379, p=0.0004

G2019S vs T1343A: Diff=-0.2640, 95%CI=-0.3132 to -0.2148, p=0.0000

G2019S vs T1343A + ATP: Diff=-0.2660, 95%CI=-0.3152 to -0.2168, p=0.0000

K1906M vs T1343A: Diff=-0.1730, 95%CI=-0.2261 to -0.1199, p=0.0000

K1906M vs T1343A + ATP: Diff=-0.1750, 95%CI=-0.2281 to -0.1219, p=0.0000

T1343A vs T1343A + ATP: Diff=-0.0020, 95%CI=-0.0512 to 0.0472, p=1.0000

**k_cat_/K_M_ Anova (Std. dev):**

Between: 7.0104 d.f. 6 Variance: 1.1684 F: 29.5277 p:0.0000

Within: 0.7518 d.f. 19 Variance: 0.0396

Total: 7.7622 d.f. 25

**Tukey HSD Post-hoc Test:**

wt vs wt + ATP: Diff=-0.5854, 95%CI=-1.1321 to -0.0387, p=0.0311

wt vs R1441G: Diff=0.8593, 95%CI=0.4210 to 1.2976, p=0.0001

wt vs G2019S: Diff=-0.2934, 95%CI=-0.7317 to 0.1449, p=0.3408

wt vs K1906M: Diff=0.8016, 95%CI=0.3244 to 1.2788, p=0.0004

wt vs T1343A: Diff=-0.3304, 95%CI=-0.7687 to 0.1079, p=0.2224

wt vs T1343A + ATP: Diff=-0.4104, 95%CI=-0.8487 to 0.0279, p=0.0755

wt + ATP vs R1441G: Diff=1.4447, 95%CI=0.8788 to 2.0106, p=0.0000

wt + ATP vs G2019S: Diff=0.2920, 95%CI=-0.2739 to 0.8579, p=0.6271

wt + ATP vs K1906M: Diff=1.3870, 95%CI=0.7905 to 1.9835, p=0.0000

wt + ATP vs T1343A: Diff=0.2550, 95%CI=-0.3109 to 0.8209, p=0.7526

wt + ATP vs T1343A + ATP: Diff=0.1750, 95%CI=-0.3909 to 0.7409, p=0.9440

R1441G vs G2019S: Diff=-1.1527, 95%CI=-1.6147 to -0.6907, p=0.0000

R1441G vs K1906M: Diff=-0.0577, 95%CI=-0.5567 to 0.4413, p=0.9997

R1441G vs T1343A: Diff=-1.1897, 95%CI=-1.6517 to -0.7277, p=0.0000

R1441G vs T1343A + ATP: Diff=-1.2697, 95%CI=-1.7317 to -0.8077, p=0.0000

G2019S vs K1906M: Diff=1.0950, 95%CI=0.5960 to 1.5940, p=0.0000

G2019S vs T1343A: Diff=-0.0370, 95%CI=-0.4990 to 0.4250, p=1.0000

G2019S vs T1343A + ATP: Diff=-0.1170, 95%CI=-0.5790 to 0.3450, p=0.9783

K1906M vs T1343A: Diff=-1.1320, 95%CI=-1.6310 to -0.6330, p=0.0000

K1906M vs T1343A + ATP: Diff=-1.2120, 95%CI=-1.7110 to -0.7130, p=0.0000

T1343A vs T1343A + ATP: Diff=-0.0800, 95%CI=-0.5420 to 0.3820, p=0.9970
