# Supplementary material for: Intramolecular feedback regulation of the LRRK2 Roc G domain by a LRRK2 kinase-dependent mechanism
Source: eLife. 2024 Dec 19;12:RP91083. doi: 10.7554/eLife.91083 (PMC11658767; doi:10.7554/eLife.91083)
Supplement: Supplementary file 1. — (a) MBP-RocCOR Michaelis–Menten kinetics as measured by charcoal-based GTPase assay. (b) GTPase activity (kobs) as measured by charcoal-based GTPase assay. (c) Cell-based phospho-Rab assays. Quantification of western blots. [file elife-91083-supp1.docx]

| **LRRK2 variant** | **K_M_ [µM]** | **k_cat_ [min^-1^]** | **k_cat_/K_M_ [min^-1^/mM]** |
| --- | --- | --- | --- |
| wt | 431 ± 23 | 1.51 ± 0.03 | 3.51 ± 0.20 |
| R1441G | 545 ± 61 | 1.70 ± 0.06 | 3.19 ± 0.29 |
| Y1699C | 587 ± 28 | 2.44 ± 0.05 | 4.18 ± 0.21 |
| T1343A | 702 ± 69 | 2.64 ± 0.09 | 3.76 ± 0.40 |

**Supplementary File 1a:** MBP-RocCOR Michaelis-Menten kinetics as measured by charcoal based GTPase assay.

| **LRRK2 variant** | **k_obs_ [min^-1^]**  **100 µM GTP** | **k_obs_ [min^-1^]**  **2000 µM GTP** |
| --- | --- | --- |
| wt | 0.08 ± 0.01 | 0.44 ± 0.12 |
| R1441G | 0.13 ± 0.01 | 0.68 ± 0.14 |
| G2019S | 0.10 ± 0.01 | 0.52 ± 0.14 |

**Supplementary File 1b:** GTPase activity (k_obs_) as measured by charcoal based GTPase assay.

| **LRRK2 variant** | **pRab10** | **pSer935** |
| --- | --- | --- |
| wt | 1.0 ± 0.0 | 1.0 ± 0.0 |
| R1441G | 8.0 ± 1.7 | 0.2 ± 0.1 |
| G2019S | 2.2 ± 0.8 | 0.9 ± 0.2 |
| I2020T | 7.3 ± 1.2 | 0.6 ± 0.3 |
| K1906M | 0.2 ± 0.1 | 1.0 ± 0.4 |
| T1343A | 2.1 ± 0.4 | 1.2 ± 0.6 |
| T1343A/G2019S | 2.5 ± 0.2 | 1.0 ± 0.4 |

**Supplementary File 1c:** Cell-based phospho-Rab assays. Quantification of Western Blots
